# *The Merchant of Venice* in Auschwitz: Taking Apart Shylock Using the SCM and BIAS Map

**DOI:** 10.3389/fpsyg.2020.602113

**Published:** 2021-01-21

**Authors:** Susan L. Knutson

**Affiliations:** Département des études Anglaises, Université Sainte-Anne, Nova Scotia, NS, Canada

**Keywords:** Stereotype Content Model, BIAS map, Shylock, Auschwitz, Shakespeare, Egervari

## Abstract

In response to *Frontiers’* 2020 Call for Papers on “Stereotypes and Intercultural Relations: Interdisciplinary Integration, New Approaches, and New Contexts,” my paper integrates the scientific study of stereotypes with a literary-theatrical exploration of stereotyping. The focus is on Tibor Egervari’s post-Auschwitz adaptation of Shakespeare’s anti-Semitic comedy *The Merchant of Venice*, with a very brief look at his related work on Christopher Marlowe’s *The Jew of Malta* and his 1998 collaboration with conductor Georg Tintner on a touring production of composer Viktor Ullmann’s and librettist Peter Kien’s one-act opera, *The Emperor of Atlantis, or Death’s Refusal*, composed in the “model” concentration camp Terezín (Theresienstadt), in 1943–1944. Egervari’s theater art critically deconstructs what he calls “the Old Jew” stereotype in specific ways highly readable using the Stereotype Content Model (SCM) and Behavior from Intergroup Affect and Stereotypes (BIAS) map. Theater performance can sometimes embody the forceful dynamic traced by the BIAS map, from cognition to affect to behavior. Egervari’s original adapation, which sets *The Merchant of Venice* in Auschwitz, reveals this dynamic clearly. My interdisciplinary study of Egervari’s theatrical-cultural work validates the SCM and BIAS map for literary studies and interprets the Shylock stereotype in the terms of those models and through the lens of Egervari’s anti-Nazi adaptation of Shakespeare’s *Merchant*.

## Introduction

The genocide of six million European Jews during the Second World War involved deliberate intensification of a pre-existent Jew stereotype. The study of stereotyping is urgent for this reason alone, but there is more. In ways that people might never have imagined, understanding stereotyping is crucial if we are to end political polarization and together build a sustainable global culture. Fortunately, stereotypes have for decades been the focus of convincing research in psychology, and are now much better understood than they were in the 1930s. The Stereotype Content Model (SCM; Fiske et al., 2002; Cuddy et al., 2008), originating in the United States and now generalized across nearly fifty countries (Grigoryev et al., 2019b), and the Behavior from Intergroup Affect and Stereotypes (BIAS) map (Cuddy et al., 2008), have identified warmth and competence as universal dimensions of individual and intergroup perception and demonstrated their social antecedents: competition predicts warmth (or its absence); status predicts competence perception. The BIAS Map describes the dynamism of cognitive stereotypes, their resulting typical affect, and the prompts and tendencies they produce for consequent behavior. This psychological research illuminates the functioning of stereotypes in our societies and provides us with tools to understand, and perhaps prevent, the harm they cause.

In theater studies too, advances have been made with respect to stereotyping. In Canada today, public performance serves to negotiate differences and identities among First Nations, between First Nations and settler/invader cultures, between French and English, and among successive waves of migrant and displaced populations (Knowles and Mündel, 2009, p. vii). In our increasingly diverse society, theater engages seriously with the problem of prejudiced and stereotypical representations of people. The coincidence of advances in social psychology and in the literary study of stereotypes suggests the timeliness of an integrative, comparative approach.

## The Jew Stereotype Shylock in *The Merchant of Venice*

When Holocaust survivor Tibor Egervari wrote and directed his anti-Nazi adaptation of Shakespeare’s *The Merchant of Venice*, setting it inside a prison inside the Auschwitz death camp, he felt that for the first time in his life, he could talk openly about the Shoah (Egervari, 2009, p. 115).^1^ At the same time, in doing this work, he made a remarkable contribution to a centuries-long conversation about the Venetian moneylender Shylock – one of Shakespeare’s most “incomparable” characters (Rowe, 1709; as cited in Drakakis, 2010, p. 1), and one who has often stolen the show. A tendency to see Shylock as the centre of the play, as opposed to Antonio, the Venetian merchant, appears in the historical record as early as 1598 when the play was listed in the Stationer’s Register as “the Jew of Venice” (Drakakis, 2010, p. 2). In her important realignment of the play’s early performance history, Emma Smith makes the point: “Almost every critic of *The Merchant of Venice* acknowledges Shylock as its most compelling figure, present in only five scenes and entirely absent from its final act” (Smith, 2013, p. 188).^2^ Shylock’s accurate insights into his own condition, his grief, anger, and pursuit of revenge, give him the complexity of a tragic hero such as Macbeth; yet he is a Jew stereotype and his play – “Shakespeare’s grand, equivocal comedy,” in the words of Harold Bloom – “is a profoundly anti-Semitic work” (Bloom, 1998, p. 171). As John Gross suggests, the anti-Semitism of the play helped to “prepare the ground” for the Nazi Holocaust, “and to that extent the play can never seem quite the same again. It is still a masterpiece; but there is a permanent chill in the air, even in the gardens of Belmont” (Gross, 1992, p. 352).

Egervari’s adaptation centers on Shylock – contrary to his own artistic and intellectual intentions: “I just wanted to give a magnificant play a proper staging,” he explains in a 2012 essay about his work (Egervari, 2012, p. 283). However, because of the weight of the war, and everything that had happened, he could not: “I felt compelled to write a new play. The idea … was not just any idea. It brought about the story I would not, I could not, speak of in any other form” (Egervari, 2012, p. 284). The story of the Shoah.

Of the staging of *Merchant* which he did *not* direct, he gives some account in the same essay. Growing up in Budapest after the war, he attended Shakespeare plays: “Nearly all […] could be seen in Stalinist Hungary, but not *The Merchant of Venice*. In the same way that the regime ‘settled’ the anti-Semitism problem by purging it from the official rhetoric, it simply eliminated any plays that could not be given a ‘correct’ Marxist interpretation from the repertory” (Egervari, 2012, p. 274). In 1956, Tibor Egervari fled Hungary to begin a new life as a refugee and theatre artist, first in France and ultimately in Canada, where he was among the founders of the theatre program at the University of Ottawa before retiring from distinguished service in 2004. He eventually saw Jean Gascon’s production of *The Merchant* in 1970, at the Stratford Festival in Ontario, when he was struck by “the Jewish questions in the play,” and first among those, by Shylock’s tragic loss of Jessica, amounting to her death, the death of his lineage, and the breaking of the commandments. Failure to understand the loss of Jessica from Shylock’s Jewish point of view impoverishes the play: “references to the jewels were interpreted as further proof of Shylock’s cupidity without noting that these ornaments pertain to Jessica and Jessica only. The dowry ornaments – for marriage to a Jew, of course – are an integral part of his daughter and therefore must die with her” (Egervari, 2012, p. 275).^3^ He was impressed as well by the symbolism of blood, recalling “the medieval persecution of Jews for supposedly using Christian blood […] from young children [....] This ignominious accusation” (Egervari, 2012, p. 275); and by Portia’s cruelty.

Egervari returned to Europe, as he recounts:

A few years later, I found myself heading a large theatre in Bussang, in the Vosges regions of eastern France – a “people’s theatre,” or *théâtre populaire* as the French call it. The *Théâtre du Peuple* was founded in 1895 by Maurice Pottecher, and until the early 1970s it performed his plays exclusively. On summer Sunday afternoons the vast wooden building with its back opened to reveal the landscape, unique in France, […] welcomed audiences representing almost every level of French society. When I took over as artistic director, my first move was to change the repertory by adding several Shakespeare plays [....] I decided to study [*The Merchant of Venice*] with a view to production. I was in for a brutal shock. I knew that Shylock had “usurped” the leading role […] but I had had no idea of the riches this usurpation was concealing. The first discovery was what I believe to be the main theme, which is the transactions or commerce […] between and of men and women [....] and the fundamental confrontation of the [medieval and modern] concepts of money.” (Egervari, 2012, pp. 275–277)

More deserves to be said about these readings of *The Merchant*, which are in tune with much of the scholarship published in the years since; however, as it happened, the production he initially imagined did not take place, but continued forcefully to evolve. Later again, resettled in Canada, Egervari composed *“Le Marchand de Venise” de Shakespeare à Auschwitz*, which focusses on the Nazi deployment of the Jew stereotype. The play premiered in French in Ottawa in 1977; it was performed in French and English in 1993, and again in 1998 (Lieblein, 2009, p. 109). Egervari himself has translated it into Hungarian (Knutson and Gray, 2012). It was, as he writes, a project which occupied him for over two decades (Egervari, 2012, p. 283).

In Egervari’s adaptation, *The Merchant* is produced inside a prison in the death camp. The fictive performance is directed by a Nazi commander, “Shylock,” who controls the prison and directs the play. One other Nazi is present: “Shylock’s” subordinate “Tubal,” stage manager and SS Officer. They are given only the names of the Shakespearean characters they embody, reflecting perhaps the fact that prisoners in Auschwitz were stripped of their names and other signifiers of identity. “Shylock” performs almost all of that character’s lines in Shakespeare’s play, and speaks also as the play’s director, training his Jewish, Gypsy, and German actors to better inhabit and communicate the “reality” of the Jew stereotype as the National Socialists promulgated it, including the notion of toxic contamination by Jewish blood, and other elements such as a stooped posture, taken from anti-Semitic Medieval engravings; he also, as a matter of course, indulges his murderous hatred of women.^4^

My paper investigates this “Shylock,” according to the terms of the SCM and BIAS Map. Significant issues include:

• the ontology of the stereotype,
• its changeable and socially determined traits and contents,
• its nature as an ambivalent Low Warmth/High Competence (LW/HC) stereotype, which prompts the behavior of passive facilitation and active harm, and
• the importance and the failure of empathy.

I argue that the SCM and BIAS Map predict the plays’ violence – even the extreme violence of Egervari’s adaptation, which pales only against the reality that was.

## Stereotype Ontology

Stereotypes are collective cognitive entities (Winiewski and Bulska, 2019) and influential players in the global cultural continuum. This is quite clear with respect to the Jew stereotype and to Shylock as an instantiation of it. Sara Coodin, in her 2017 monograph, writes eloquently about the ways that the Shylock character, as a stereotype, causes harm to people:

[A] bitter truth about *The Merchant of Venice*: Shylock is a figure closely bound up not only with the fictionalized landscape of anti-Semitism on the page but also with its lived history off of it. *The Merchant of Venice* has furnished notable turns of anti-Semitic phrase, foremost among them the term “Shylock” which has come to describe a cut-throat type of Jewish profiteer. This play and its Jewish moneylender [tend] to bleed messily off the page into historical actuality [....]. (Coodin, 2017, pp. 4–5)

She gracefully acknowledges Cecil Roth, who made the same point almost ninety years ago, in the decade leading up to the Second World War:

That Shylock was a sheer figure of Shakespeare’s imagination, there has never been any doubt. Yet this figment has acquired an objective reality more vital than that of most creatures of flesh and blood. His actions are still a byword, his name is a reproach, and his unfortunate co-religionists actually taxed with his reputed misdeeds. (Roth, 1933, p. 148; cited in Coodin, 2017, p. 5)

Coodin’s monograph explores the rich Judaic exegesis of the Jacob narrative as deployed by Shylock, in Shakespeare’s text, but she acknowledges the social and cultural influence of the equally legible Jew stereotype:

Jews remain inextricably if unwittingly bound to Shakespeare’s fictional moneylender because of the ways in which he and Jessica have helped construct Jewishness in the popular imagination. In looking at Shylock and being confronted with turns of phrase from the play which, like Shylock himself, have become bywords in vernacular speech, modern Jews glimpse a reflection of their ethnographic identity through the distortive lens of interpolated stereotypes that have played a significant role in shaping cultural perceptions of Jews over time. (Coodin, 2017, p. 5)^5^

Since the war, the topic of Shylock and anti-Semitic and anti-Judaic stereotyping has received significant attention. Outstanding monographs dedicated to this work include John Gross’s *Shylock: A Legend and Its Legacy* (1992), James Shapiro’s (1996), *Shakespeare and the Jews* (1996), Janet Adelman’s (2008), *Blood Relations: Christian and Jew in* The Merchant of Venice (2008), and David Nirenberg’s (2013) *Anti-Judaism: The Western Tradition* (2013). Schools of thought across the Humanities – New Historicist, Psychoanalytic, Post-Colonial and more – have worked to understand the charisma of the character Shylock and the uses to which he has been put.

A related effort works to untangle the roots of Shakespeare’s Shylock from a wide range of cultural and social models, including Christopher Marlowe’s *The Jew of Malta*, and early modern Europe’s violent religious and economic conflicts. It is thought that both Marlowe and Shakespeare drew on “older (non-Jewish) European traditions of hate literature” (Orkin, 1998, p. 196), and that anti-Judaic violence was fueled for centuries by stereotypes assigning to Jewish people a wildly exaggerated competency alongside derogatory traits as bizarre as male menstruation, cannibalism, and child murder (Bildhauer, 2020). These have a long association with blood, which makes its way importantly into Shakespeare’s *Merchant* and Egervari’s adaptation. Nirenberg (2013) and Freinkel (2002, pp. 115–158; 237–291) have made crucial contributions in this field, deciphering the figure of the Jew in the discursive tradition and exposing the damage done by a current of anti-Judaic Christian theology, and especially by Martin Luther.

With respect to theater history, Emma Smith has recently overturned widespread critical assumptions “about the play’s Elizabethan context [that] do not stand up to close investigation. Recent criticism has used a partial and anecdotal version of theatrical and social history to reify Shylock’s ‘original’ cultural and ethnic Jewishness” (Smith, 2013, p. 188). She traces the origins of the physical features thought to signify the stereotyped Jew on the stage, including perhaps a red beard and “bottle” or hooked nose: fictions all. The paucity of early modern references to specific visual signifiers of Jewishness suggests that Portia’s question in the courtroom, “Which is the merchant here, and which the Jew? (4.1.171) is a real one” (Smith, 2013, p. 201).^6^ Widespread claims that “Shylock draws on an existing and negative literary and theatrical caricature of Jewishness” are contrary to fact: “Jewish characters in drama before *The Merchant of Venice* are rare and sufficiently diverse to compromise any claim that they constitute an available stereotype” (Smith, 2013, p. 203). Furthermore, “the history of marked Jewish characters on the stage before Shylock does not support the assumption that Elizabethan audiences were primed to expect a wicked stereotype, or even that such a stereotype can be traced (Smith, 2013, p. 208). She suggests that in addition to anti-Judaic discourses, there were other factors at play when Shylock was created: issues also familiar to us today, such as immigration and xenophobia.

It might seem to be simple common sense to break apart naive mimetic association between real Jews and the stereotype; yet, John Drakakis, discussing a 1990 essay by Stephen Greenblatt, suggests that the “shift in much of contemporary criticism of *Merchant*, from an essentially mimetic commitment to social realism, to larger questions of representation, is a recent one” (Drakakis, 2010, p. 29). Drakakis (2010) suggests that “stage representations that are not validated by actual social example, such as those of Barabas or Shylock, straddle an important conceptual divide,” and he goes on: “such representations form part of that *otherness* against which communal identity asserts itself,”^7^ that which “cannot be domesticated” (p. 27). He cites Jean-François Lyotard:

One converts Jews in the Middle Ages, they resist by mental restriction. One expels them during the classical age, they return. One integrates them in the modern era, they persist in their difference. One exterminates them in the twentieth century.” (Lyotard, 1990, p. 23; as cited by Drakakis, 2010, p. 29)

To argue that Jews are what “cannot be domesticated” potentially returns us to a naive mimetic realism that has been rightly problematized. It is necessary to better understand what stereotypes actually are, how they are formed, of what they consist: their being, their ontology – a task undertaken by scientific research.

In their 2019 paper on discrimination against immigrants, Grigoryev et al. (2019a, closing paragraph) integrate ultimate (functional) and proximate (sociofunctional) models of discrimination to better understand prejudice and possibly to serve as a basis for future policy development. The ultimate (functional) explanation of prejudice suggested by the Evolutionary-Coalitional model encompasses the notion of “what cannot be domesticated” – but it has nothing to do with Jews or with any other group of actual people. The model proposes that “Us” versus “Them” dynamics are a product of “the evolutionary core of intergroup relations”:

[Over evolutionary time a] cognitive mechanism […] evolved to detect coalitional alliances via the categorization of the social world into “Us” versus “Them”; this is what ultimately predisposes humans to discriminate in favor of their ingroup and against the outgroup. For the human mind, ethnicity, cultural group, or race is simply one historically contingent subtype of coalition because through a long human story, they have been an ecologically valid predictor of people’s social alliances and coalitional affiliations. (Kurzban et al., 2001; as cited in Grigoryev et al., 2019a, Theoretical Framework)

This model dissassociates stereotypes from human beings, potentially providing an explanatory mechanism for the denial of humanity to some, and its retention for others.

Stereotypes are cognitive realities, but they are not people. In their 2019 paper on stereotype content as collective memory, Mikołaj Winiewski and Dominika Bulska review scientific understandings of the reality status of stereotypes: “stereotypes exist as cognitive structures, such as schemas (Fiske and Linville, 1980), prototypes (Brewer et al., 1981), or exemplars (Smith and Zarate, 1990).” They explain:

For several decades psychology was mainly interested in the process of stereotyping, and few studies focused on the content of stereotypes – their specific traits (Fiske et al., 2002). Some scholars, however, noted that aside from the individual perceptions of groups and group members, stereotypes are shared across communities or entire societies, and are thus a collective entity – a part of shared knowledge (Ashmore and Del Boca, 1981; Devine, 1989). Collective stereotypes and intergroup stereotyping processes – how ingroup members perceive other groups – largely shape relationships between groups, but these relationships also seem to be, at least in part, a source of stereotypical content. (Winiewski and Bulska, 2019, p. 2)

As collective cognitive entities in society and a part of shared or so-called knowledge in real time, stereotypes are actors in a dynamic, two-way relationship with material social realities. Current research describes what stereotypes are: their contents, traits and rapport with social structures and histories. It is shocking to think that powerful perceptions such as those of warmth and competence are not in fact triggered by actual loving kindness or real competence, but are in fact produced by historical, social, collective circumstances such as competition for life resources or inherited social class, but that is what the research suggests. My reading of Egervari’s adaptation of *The Merchant of Venice* looks at the stereotype features or symptoms, rather than the interplay with actual Jewish people, such as Coodin explores in her beautiful exegesis of Shylock’s Midrash-inflected moral invocation of the patriarch Jacob. I am interested in the stereotype as such.

Shakespeare’s *Merchant* has been a vector for sharing the Shylock stereotype across communities and over time, and never more than in the twentieth century. Shylock played a role in the Nazi transmission and perpetuation of the Jew Stereotype. The relation between theater performance and the destructive reality of the stereotype in the years leading up to and during the Second World War was tangible and direct.

Recent scholarship focused on theatrical production has explored the complex place of *The Merchant* during those years. Zeno Ackermann’s “Shakespearean Negotiations in the Perpetrator Society: German Productions of *The Merchant of Venice* during the Second World War” (Ackermann, 2012, pp. 35–62) traces the performance history:

Since the end of the eighteenth century the play had always held an important place in the German Shakespeare canon. According to the performance statistics published in the yearbooks of the German Shakespeare Society, *The Merchant* ranked first among Shakespeare plays in 1927; it held third place in 1928, 1929, and 1931, and fourth place in 1932. By 1941, however, the number of performances would reach an all-time low of three shows, staged in a provincial theatre in Bohemia (annexed by the Reich in consequence of the 1938 Munich Agreement): in the listings for that year *The Merchant* held twenty-first place, just ahead of *The Merry Wives of Windsor*. There were still nine new productions during the 1933-4 theatre season, but numbers dropped to usually one or two for the following seasons (Eicher 304). Thomas Eicher attributes these declining numbers to systematic interventions by the administration, claiming that the play was in effect “stopped.”^8^ (Ackermann, 2012, pp. 35–36)

The almost complete blackout of *The Merchant* in the early years of the war, followed by a few tightly controlled spectacles engineered and approved by the Nazi Ministry of Propaganda, demonstrate that “as a figure or as a stereotype, Shylock certainly was an important reference point, both for the self-image of the National Socialists and for their anti-Semitic propaganda.” (36)^9^ On the other hand, the stance of the Nazi bureaucracy and of cultural makers was not as straightforward as we might have thought; Ackermann characterizes it as “twisted” (Ackermann, 2012, p. 35).

He goes on to prove that the demonized Jew of the earlier years became a source of anxiety once the war and the camps were underway. The content of the stereotype was changed, to re-create Shylock as pathetic, comic, disgusting – played to reassure a public that was aware of the Holocaust that the enemy had been expunged and was now harmless. He suggests, in other words, that the Shylock stereotype was intentionally re-engineered to perform another kind of cultural work: “In the case of *The Merchant* the most important propaganda task was not to demonize but to downsize Shylock” (Ackermann, 2012, p. 50).^10^ Apparently, the Nazis of that era perferred a silly, clownish Shylock, with all tragedy removed: a *Commedia dell‘arte* Shylock, or a fairy tale bogeyman, who – horrifyingly – according to the review of Lother Müthel’s Nazi-approved production at the Burgtheater in Vienna in 1943, penned by one Siegfried Melchinger – “just like the witch, will finally have to be shoved into oven” (Melchinger, 1943; cited by Ackermann, 2012, p. 55; trans. by Ackermann).

The Nazi modulation of the stereotype from a fearsome and dangerous enemy to a figure of harmless contempt – thereby attempting to normalize their genocidal acts – is consistent with the SCM’s explanation that although stereotypes may persist, their contents can change over time and across societies and cultures.^11^ A similar modification of the Jew stereotype might be reflected in the fact that, while the intelligence of Jews is a relatively stable feature of the stereotype in recent centuries, during the Roman empire, Jews were considered to be dull-witted, in correlation with impoverishment and lack of education in the Jewish community at that time (Daniel, 1979; as cited in Winiewski and Bulska, 2019). Shakespeare’s Shylock, however, is intelligent; it would be hard, although not impossible, to perform the character otherwise. Ackermann asks: “How much ‘Angst’ did Shakespeare’s profoundly ambivalent figure of a thwarted Jewish avenger inspire in the proponents of an eliminatory anti-Semitism?” (Ackermann, 2012, p. 38). He concludes:

Shylock had acquired profoundly ambivalent significations and functions: as a figuration of difference he simultaneously unsettled *and* ratified the fantasies of the Nazis. This is why the administrators of National Socialist cultural policy were so cautious about allowing Shylock to appear on the stage – and, at the same time, it is why they were so eager to make him serve their ends. (Ackermann, 2012, p. 46)

Egervari’s *Merchant* adaptation reformulates this very paradox and with it, the eagerness of a Nazi Commander who is driven to put on the play, to direct its every meaning, and to trust only to himself the critical task of portraying Shylock.

## The SCM and Shylock: An ICY, Ambivalent Stereotype Negates Empathy

This section discusses those scenes from Egervari’s adaptation which reproduce the text of Shakespeare’s play – scenes which, in several respects, map very well onto the SCM, which links warmth and competence – predicted respectively by competition and status – with shifting social structures (Fiske et al., 2002; Cuddy et al., 2008).^12^

The SCM and BIAS map illuminate how, under socially stressful conditions, the low warmth/high competence (LW/HC) stereotype and the envious prejudice it generates can play a role in outbreaks of so-called ethnic cleansing and genocide. Amy Cuddy, Susan Fiske and Peter Glick discuss envious prejudice and the history of the Jew stereotype (Cuddy et al., 2008, pp. 127–129):

[T]he BIAS map may help us to understand “why envied groups are often tolerated but later attacked, particularly under [socially stressful] conditions that convert envy into anger…. The dynamics of envious prejudice demand further study because this type of prejudice may help to explain the most extreme form of intergroup hostility, genocidal attack.” (Cuddy et al., 2008, p. 129)

In 1543, Martin Luther influentially contributed to social stress by arguing that Jews’ familiar religious bond with their God “fleeced” Christians (Luther, 1971; as cited in Drakakis, 2010, p. 18). In the same tract, Luther “posited a specific connection between Jews and usury, although it emerged as part of a more general expression of moral outrage and resentment at the Jewish claim to be God’s chosen people” (Drakakis, 2010, pp.16–17). For Shylock, this means that the social antecedents of his stereotyping (and so, of his destruction) are his integration into the Venetian financial sector, and, simply, his cultural and ethnic identity as a Jew.

Research demonstrates that whereas both warmth and competence are core dimensions of social perceptions, “warmth judgments are primary, both in the sense that warmth is judged before competence and that warmth judgments carry more weight in affective and behavioral reactions” (Cuddy et al., 2008, p. 89).^13^ This is particularly important because the absence of warmth perception can and sometimes has led to dehumanization and active harm, including genocide. With respect to preventing this kind of harm, Thomas Pettigrew and Linda Tropp have tested intergroup contact theory’s basic contention that contact typically reduces prejudice by increasing knowledge about the outgroup, reducing anxiety about contact, and “increasing empathy and perspective taking.” They found that “the mediational value of increased knowledge appears less strong than anxiety reduction and empathy” (Pettigrew and Tropp, 2008, p. 922).

Shakespeare’s play explores the question of empathy in some detail in scenes involving Shylock and the ingroup composed of Venetian Christian merchants and aristocrats. When Antonio and Bassanio visit Shylock in order to borrow money, their conversation exhibits characteristic features cued by the ambivalent LW/HC stereotype, namely behavior combining passive facilitation and active harm. As noted above, this behavior can break down into dangerous violence in socially stressed conditions (Cuddy et al., 2008). The play dramatizes the dynamic linking of social antecedents to behavior: competition → coldness → consequences.

Shylock initially responds to the Venetians’ request by accurately describing the mixed behavior to which he has been subject in Venice as a financially successful resident alien Jew: they appeal to him for financial services, yet spit upon him in the street, and call him a dog. The Venetians’ specific behaviors accord with those predicted by the SCM and BIAS map as a consequence of the high competence/low warmth stereotype: passive facilitation alongside active harm (Cuddy et al., 2008). In one of the plays most extraordinary and well-known scenes, passive facilitation accompanied by active harm is spelled out:

ANTONIO
Well, Shylock, shall we be beholding to you?
JEW
Signior Antonio, many a time and oft
In the Rialto you have rated me
About my moneys and my usances.
Still have I borne it with a patient shrug,
For sufferance is the badge of all our tribe.
You call me misbeliever, cut-throat dog,
And spit upon my Jewish gaberdine,
And all for use of that which is mine own.
Well, then, it now appears you need my help.
Go to, then, you come to me, and you say,
“Shylock, we would have moneys.” You say so.
You, that did void your rheum upon my beard
And foot me as you spurn a stranger cur
Over your threshold, moneys is your suit.
What should I say to you? Should I not say,
“Hath a dog money? is it possible
A cur can lend three thousand ducats?” Or
Shall I bend low and in a bondman’s key,
With bated breath and whispering humbleness,
Say this: “Fair sir, you spat on me on Wednesday last,
You spurned me such a day; another time,
You called me dog; and, for these courtesies,
I’ll lend you thus much moneys.”
(1.3.101–124)

Interpreting this scene through the lens of the SCM, we can affirm that the stereotyped subject articulates in detail the behavioral consequences of his perceived status as a highly competent and very cold person in the eyes of his Venetian interlocutors. He specifies the contradiction inherent in Antonio’s passive facilitation and active harm. Antonio, however, remains committed to the abusive behaviors that flow from his prejudiced beliefs.

Another feature of the SCM that is captured by Shakespeare’s play involves the primacy of warmth and the potentially transformative role of empathy and perspective taking. The negotiation of the contract between Shylock and Antonio involves a three-way conversation and interchange of views that could have moderated the evolving hostility. Antonio unkindly and recklessly refuses an offer of “kindness” from Shylock, who in turn suggests a contract that is anything but kind. In Shakespeare’s English, the word “kind” is layered and polyvalent, suggesting not only niceness, or generosity, as the word is used today, but also including the etymological sense of kinship,^14^ or shared ingroup status, which broadly speaking implies seeing things from a shared perspective. It seems reasonable to suggest that the Shakespearean sense of “kindness” is not far from what we mean today by empathy.

Following Antonio’s rebuff, the exchange ends with the contract involving the pound of flesh:

JEW
Why, look you, how you storm.
I would be friends with you and have your love,
Forget the shames that you have stained me with,
Supply your present wants and take no doit
Of usance for my moneys, and you’ll not hear me.
This is kind I offer.
BASSANIO
This were kindness.
JEW
This kindness will I show.
Go with me to a notary, seal me there
Your single bond, and, in a merry sport,
If you pay me not on such a day,
In such a place, such sum, or sums, as are
Expressed in the condition, let the forfeit
Be nominated for an equal pound
Of your fair flesh, to be cut off and taken
In what part of your body pleaseth me.
ANTONIO
Content, in faith: I’ll seal to such a bond
And say there is much kindness in the Jew.
(1.3.133–149)

The passive facilitation cued by the ambivalent LW/HC stereotype can take the form of mercantile patronage, and such is the case here. Shylock recognizes the mixed consequences of his outsider stereotyped status, and his daughter Jessica, too, acknowledges her outsider status. She, however, “may still identify with aspects of the societal reference group” (Cuddy et al., 2008, p. 78), whereas Shylock is faithful to his “tribe,” to use his word. When he initially points to Antonio’s contradictory behavior, his words are an appeal to reason and therefore, to some extent, an invitation to see things from his point of view. Rebuffed, Shylock retaliates.

Empathy and perspective taking counteract negative stereotyping (Pettigrew and Tropp, 2008) and its consequent prompts and effects; therefore, this scene of contract negotiations can be interpreted to mean that if Antonio had listened with any degree of empathy to Shylock or to Bassanio (“This were kindness”), rather than scorning a momentary potential for mutual perspective taking, events might have played out differently. The negative consequences might not have been triggered. When the tables turn again, and appeals for empathy are made on Antonio’s behalf, Shylock has been emotionally devasted by his daughter’s deception, her elopement with one of Bassanio’s associates, and her theft, all compounded by further harassment in the streets. In Act 3, he rehearses his dehumanization, refuses to offer empathy to Antonio, and rejects Salarino’s reasonable point. His powerful rhetorical volley expresses his anger:

SALARINO
Why, I am sure, if he forfeit, thou wilt not take his flesh: what’s that good for?
JEW
To bait fish withal; if it will feed nothing else, it will feed my revenge. He hath disgraced me, and hindered me half a million, laughed at my losses, mocked at my gains, scorned my nation, thwarted my bargains, cooled my friends, heated mine enemies, and what’s his reason? I am a Jew. Hath not a Jew eyes? Hath not a Jew hands, organs, dimensions, senses, affections, passions? Fed with the same food, hurt with the same weapons, subject to the same diseases, healed by the same means, warmed and cooled by the same winter and summer as a Christian is? If you prick us do we not bleed? If you tickle us do we not laugh? If you poison us do we not die? And if you wrong us shall we not revenge? If we are like you in the rest, we will resemble you in that. If a Jew wrong a Christian, what is his humility? Revenge. If a Christian wrong a Jew, what should his sufferance be by Christian example? Why, revenge! The villainy you teach me I will execute, and it shall go hard but I will better the instruction. (3.1.46–66)

Empathy continues to be at issue in the courtroom scene in Act 4: appeals for kindness and perspective are tossed back and forth. Portia, in her cross-dressed role as the “young doctor of Rome” (4.1.151–152) delivers a celebrated set speech in praise of mercy:

PORTIA
Then must the Jew be merciful.
JEW
One what compulsion must I? Tell me that.
PORTIA
The quality of mercy is not strained:
It droppeth as the gentle rain from heaven
Upon the place beneath. It is twice blest:
It blesseth him that gives and him that takes.
’Tis mightiest in the mightiest; it becomes
The throned monarch better than his crown.
(4.1.178–186)

Portia frames her legal arguments by the Christian theological concept of mercy, which she subsequently identifies with charity, Latin *caritas*, or God’s unbounded love (4.1.257). This is interesting in relation to Luther’s implication about competing for that love, as there is no point in competing for what is unlimited. *Caritas* plausibly directs us away from prejudice and toward the variety of Christian humanism that saw all human beings as equal; however, theology no doubt takes my argument too far afield. The critical point is that, however it is named, empathy and perspective taking – and reason – are refused on all sides, and a dangerous and angry dehumanization of a stereotyped subject is unleashed. The play acts out what happens when empathy is denied. It shows the workings of social prejudice based on stereotyped intergroup and interpersonal perceptions, and it shows how the destructive dynamic could have been, but was not, interrupted by empathic perspective taking.

Scholars and artists, including Egervari, have vehemently drawn out the profound failure of empathy in this play, noting that in spite of her beautiful words, Portia not only refuses empathy, but is the most cruel:

Portia’s – or Shakespeare’s – behavior toward Antonio is in fact as cruel as anything Shylock does. The scene is drawn out excruciatingly, and its theatrical power has much less to do with the quality of mercy than with the pleasure of sadism on the one hand and revenge on the other. (Orgel, 2003, p. 159)

Stephen Orgel points out that the “old law that Portia suddenly invokes allows … for *us* to have *our* revenge”:

[T]he old law is a secret, in effect an *ex post facto* law, which applies only to Shylock, and has been invoked – indeed invented – solely to put him at the mercy of the court. This is a striking example of the play’s tendency toward overkill, because the forgotten law is Shakespeare’s invention, appearing in none of the sources, and quite unnecessay to the plot. (Orgel, 2003, p. 160)

Egervari, too, exercises what might be called overkill: his “Portia,” a cultivated and empathetic Gypsy woman, gives the “quality of mercy speech” in the costume of Hiter, moustache etc., and furthermore is awarded the most brutal of all the many deaths, her head forced into a bucket of burning coals. But there is nothing even superficially soothing about Egervari’s play.

Cuddy, Fiske and Glick point out that negative behavior associated with ambivalent social stereotypes works to maintain the social status quo:

Despite their ambivalent content, envious and paternalistic stereotypes still function to maintain the status quo and defend the position of societal reference groups (also see Jost et al., 2001 for this argument). Further, as a form of cross-dimensional ambivalence (MacDonald and Zanna, 1998), these combinations are psychologically consistent for perceivers. [who] can imagine a group as being warm but incompetent or as competent but cold without experiencing the psychological tension that is classically assumed (e.g., by Freud) to be integral to ambivalence. [T]his has obscured the true nature of important forms of prejudice. These include the oldest form of prejudice – sexisim, which has long fostered inequality through paternalism (Glick and Fiske, 1996; Jackman, 1994) – and the most severe form of prejudice – genocidal hatred, which is most commonly directed toward successful, envied minorities (Glick, 2002, 2005). (Cuddy et al., 2008, p. 76)

Prejudice and discriminatory practises against the Jewish minority are represented as the legal normal in Shakespeare’s dramatized version of Venice, and the plot supports this discriminatory status quo, with the expiry of a Jewish family line, the confiscation of Jewish material wealth by the Christian majority, and Shylock’s forced conversion to Christianity; all can be interpreted as positive outcomes. The play’s conclusion as a Christian, if not quite comedic, resolution, can be read as mercy: Shylock’s life is spared, and, with some unclarity, half of his worldly goods will go to his daughter’s husband, Lorenzo, rather than to Antonio. Venetian society is stabilized, and three marriages symbolize the continuity of the upper class. For such reasons, this play is classically termed a comedy. Today, we might term it a thought experiment in social psychology.

Shakespeare’s *Merchant* reassures the ingroup, composed of the early modern Christian elite, and it shores up the prejudiced, anti-Semitic status quo. Egervari’s adaptation pushes this cruel, comedic trope to the extreme. Is it possible to refer to SS control of the Auschwitz death camp as a status quo? Following “Shylock’s” killing, “Tubal” murders the actors one by one, “Jessica” going last in a scene of sexual humiliation. The light goes out on a solitary man, examining a diamond solitaire – seated calmly, smoking a cigar, listening to a waltz. In Egervari’s play, psychological tension is brought to a boil, not by the stereotype *per se*, but by its pre-eminence in the world of the play, located inside a prison, inside Auschwitz. Egervari’s “Merchant” alerts, shocks, and warns, but in no way reassures.

## Anti-Nazi Theater: Tibor Egervari “Shylock” in Auschwitz

For over two decades, Egervari wrote, adapted and directed theater projects staging the deconstruction of the stereotype of the “old Jew” (to use his term). “*Shakespeare’s* The Merchant of Venice *in Auschwitz*” is the only one to survive as a play, but there were companion works. His 2011 adaptation of Marlowe’s *The Jew of Malta* deconstructed the Jew stereotype by using a dresser, inspired by Japanese theater: a beautiful young female actor gradually transformed, using make-up and costume, into a hideous “old Jew” – under the audience’s gaze, in full sight. The stereotype is not the person. Earlier, in 1998, he collaborated with renowned conductor Georg Tintner on a touring production of composer Viktor Ullmann’s and librettist Peter Kien’s one-act opera, *The Emperor of Atlantis*, *or Death’s Refusal*, composed in the “model” concentration camp Terezín (Theresienstadt), in 1943–4. Costuming and theatrical transformations dramatically expose the reality that the human beings are Jews, in the costumes of the *Häftlinge*; the Emperor singing the aria is a Jew. Egervari’s directorial style draws on intensifying techniques learned from Japanese Noh and the theater work of Luigi Pirandello, Antonin Artaud, Bertolt Brecht, Jerzy Grotowski, and others (T. Egervari, personal communication, Ottawa, June 24, 2015). As a survivor and an artist, his ongoing implication with the trauma of Auschwitz is expressed through theater, and his “Merchant” is an extraordinary accomplishment.

Egervari’s “Merchant” harnesses the power of Shakespeare’s, following through, as Shakespeare does, with Shylock’s defeat. The difference is that Egervari’s “Shylock” is a Nazi commander/actor-director who is staging the play in order to prove the reality of the toxic Jew stereotype that justifies his mass murders. The dramatic deconstruction of the anti-Semitic stereotype “Shylock” is founded in the first place on the theater basics of costuming and acting, and the play’s success as an anti-Nazi work is founded on theater’s power to change minds.

How is this work readable using the SCM and BIAS map? As discussed above, the SCM identifies warmth and competence as universal dimensions of social perception, and posits that warmth and competence judgments are based on the socially contingent and temporally mutable antecedents of competition and status. “People viewed as competitors are judged as lacking warmth, whereas people viewed as non-competitors are judged as warm; people viewed as high status are judged as competent, whereas people viewed as low status are judged as incompetent” (Cuddy et al., 2008, pp. 92–93). While the two underlying dimensions of warmth and competency are universal, the stereotype traits themselves can and do change.

The SCM exposes the disconnect between real individuals and the stereotypes that are their unwelcome doubles. It exposes the fact that the all-important warmth of the stereotype/perception is not determined by actual warmth but by perception of social structural competition, and competency is not determined by actual competence but by social status, which may be undeserved. Complex histories of conflict and collective memory underlie both competition and status, yet there is an actual disconnect and non-correspondence between the motivating conditions for the stereotype *per se* and any given person or group to whom that stereotype, with all of its effects, is applied. Stereotypes are real, but they are not real people. Nonetheless, in Egervari’s “Merchant” it is the mission and the passionate intention of the Nazi commander/actor-director “Shylock” to prove the opposite.

The play opens in the dark, inside a locked prison in the death camp. The first character to speak is the Nazi lieutenant commander who is directing Shakespeare’s *Merchant*, and playing Shylock. He sees the play as a vector for his message (i.e., the stereotype), and with respect to his choice to play the hated Jew, he will explain. The SCM can offer another reason: his appropriation of the role reflects the envy he feels toward the Jew stereotype because of his prejudiced belief in the exaggerated competency of the Jews as powerful conspirators (Cuddy et al., 2008). His casting of the other roles also reflects this belief: “Tubal” is an SS Officer; two gypsy women and a common criminal *kapo* play Portia, Nerissa, and Launcelot Gobbo, respectively. The Venetian elite, “Antonio,” “Bassanio,” “Gratanio” and the others, are real Jewish prisoners, and “Jessica” is a German actress. All the Jewish characters are played by so-called “pure Aryans” (Egervari, 2009, p. 143). What reason for this reversal? asks “Jessica.” “Shylock” replies:

Do you honestly believe those Yids would project the image I wish to show of them?
We’re the only ones who can unveil their true identity.
(Egervari, 2009, p. 144)

“Shylock” insists on offensive and derogatory terminology at all times to refer to the Jewish characters (but not to the actual Jewish prisoners), while he crucially affirms that the stereotype is the true Jewish “identity.” His envious need to assert himself in relation to the Jew stereotype, suggested by his initial decision to play Shylock, is exposed as well by his belief that he alone can reveal, through the power of theatrical performance, the truth of the prototypical exemplar:

SHYLOCK Jessica!
JESSICA Good morning, sir.
SHYLOCK I am your father!
JESSICA Yes, in the play, but …
SHYLOCK We are in the play! Listen to me very carefully. As my assistant has told you, this concerns a project of the highest importance. You have been chosen for your talent, I’ve seen you perform; because you’re a pure Aryan, like me, like Tubal, and you are here on official assignment. It’s our duty to unveil the true face of this enemy race, which the Führer has defined as a moral plague worse than the black plague of early times. Have you read *Mein Kampf*? (Egervari, 2009, p. 143)

The first principle affirmed by the Nazi criminal theater director is the supposed identity between the Jew stereotype and real Jewish people, something, of course, that is completely disproven in detail – if proof were necessary, and it seems unfortunately that it is – by current research in social psychology, and especially by the SCM and BIAS map, which illuminates the nature of all stereotypes. In the world of this play, however, the Nazi commander believes in his ontological fallacy and supports his argument using Shakespeare’s *Merchant.* As the play proceeds, he also refers to well-known works of anti-Semitic propaganda including *Mein Kampf* and *The Protocols of the Elders of Zion*. Believing that the Jewish prisoners would subvert his message in the projected performance, he has given the Jewish roles to the Nazis, and the Gentile and elite Venetian roles to the prisoners.

As a method actor in the tradition of “good’ theater schools of the 1930s” (Egervari, 2009, p. 122), “Shylock” insists that the cast remain in their roles at all times. He himself evolves gradually into his character, a fatal transformation that is visually represented on the stage:

• He is “in his thirties, dressed in sports clothes – black shorts, white vest – a towel around his neck” p. 117;
• “lying on his stomach [for a massage] … “covered with a large towel” p. 121;
• in his bathrobe and has covered his head” p. 122;
• “practises a Hasidic song as he glues on his beard” p. 154;
• “costume and makeup are almost complete” p. 155;
• “rage bordering on madness” p. 164;
• “his prostration” p. 166;
• “Shylock is in his dressing room” p. 171;
• “Enters Shylock, who now appears as a Hasidic Jew” p. 171.

At the end, he achieves an appearance that demonstrates – or so he wishes – the dangerous reality of the Jew stereotype. However, the very fact that the audience and the other players have seen his theatrical transformation, his costuming, and his blocked and choreographed actions exposes the stereotype as a theatrical role and undermines his intention.

As I have mentioned, choreographed costuming is also a necessary aspect of stereotype deconstruction in Egervari’s *The Jew of Malta*, where a “dresser” transforms a beautiful young female actor into Marlowe’s vicious and visceral representation of the Jew stereotype. Egervari’s production of *The Emperor of Atlantis* also featured a complex staging of costume changes, setting, and music. (Van Vlasselaer, 1998)

In Egervari’s “Merchant,” the transformation of the unnamed German actress into the Jewish daughter and heiress “Jessica” involves the transference of the play’s father–daughter conflict into her dressing room and onto her physical body. The real actor loses her identity, so recalling the many inflictions intended to destroy the identities of the incarcerated people in the Nazi concentration camps. The world of the play and the world of the Holocaust become entangled.

SHYLOCK [to “Jessica”] Has no one ever told you that it is the soldier’s main duty to study the enemy? You definitely need to read *Mein Kampf*. She has always longed to leave her father whom she despises, just like she despises her Jewish condition. She secretly read books which her father forbade her to read. We have hidden a cross, some books on mythology, and the Gospels in your dressing room. You are to read them in secret, and if I, Shylock, catch you in the act, you will be beaten for it.
JESSICA Beaten … for real …? But you have no right. I’m German, and Aryan, a Christian.
SHYLOCK Not quite anymore, and not yet.
JESSICA I don’t understand, and anyway, under these conditions, I’m afraid I will have to refuse the part.
SHYLOCK I would like you to fully understand our situation: this has nothing to do with your little theater in the provinces, and you were not hired, you were conscripted! Furthermore, we are at the front. Our enemies are in front of you, and you will not be able to leave this place before we get to the final solution.
(Egervari, 2009, p. 146)
[….]
SHYLOCK [to “Launcelot”] You see her, she’s a Jewess now. This morning, she was still an Aryan, but now, she’s a darling, stinking Yid. The magic of theater. (Egervari, 2009, p. 168)

## Cognition to Affect to Behavior: The Dynamics of “Minds Transfiguring Together”

There is more to Egervari’s magical world. To unpack what happens, we must move to the far right edge of the BIAS map, and the hypotheses involving the emotional, behavioral and attributional consequences of stereotype perception. Moving from antecedents (warmth and competence) to consequences, Cuddy, Fiske and Glick ask how warmth and competence judgments affect the ways that targets are treated. They propose that perceptions of warmth and competence elicit predictable, differentiated patterns of social emotions, behaviors and attibutions. People perceived as incompetent and not warm elicit contempt and disgust, and those perceived as competent but cold, e.g., in this case, Jews, elicit envy (Cuddy et al., 2008, p. 102). Cognition of the ambivalent LW/HC Jew stereotype would be typically followed by its “distinct emotional profile which elicits a discrete pattern of behavioral reponses, namely passive facilitation and active harm” (Cuddy et al., 2008, 107).

Cognition → Affect → Behavior
Low Warmth/High Competence Stereotype → Envy → Passive facilitation and Active Harm

A significant lack of warmth, particularly in stressful social conditions, could create “a relatively urgent need to react,” and perhaps to react violently (Cuddy et al., 2008, pp. 110, 112). This dynamic sequence with its cues for action may be supported by neurological prompts, although the researchers make the important point that “representations in the brain do not mean that prejudice is hard-wired and evitable: social context affects neural responses, naturally” (Cuddy et al., 2008, pp. 135–136).

Theater is an art of action-reaction and does not neglect this powerful dynamic, which is familiar to literature across the genres, from detective novels and love stories to Sophocles. “Shylock’s” theatrical transformation into the worst possible version of the Nazi Jew stereotype – all taking place inside a prison inside Auschwitz – plausibly triggers dynamic, violent action that is readable using the SCM and BIAS map.

Envy is the emotion that the SCM predicts will follow from a LW/HC stereotype. It is interesting that in Shakespeare’s *Merchant*, Antonio doesn’t express envy. He expresses hatred toward Shylock, as I have shown, and Shylock, of course, hates Antonio (e.g., 1.3.38). However, anger has been shown to mediate the link from envy to activation (Cuddy et al., 2008, p. 116); furthermore, “people are loath to admit envy because it implies a deficit in the self or ingroup” (Cuddy et al., 2008, p. 104). In the text, Antonio asserts his superiority, in spite of the fact that he lacks money and finds himself obliged to go to the rich Jew for help.

When speaking with the Duke about the failure of appeals to Shylock to accept payment rather than a pound of Antonio’s flesh, as specifed in his contract, Antonio goes so far as to suggest that he himself is the target of Shylock’s envy:

since … no lawful means can carry me
Out of his envy’s reach, I do oppose
My patience to his fury, and am armed
To suffer with a quietness of spirit,
The very tyranny and rage of his. (4.1.7–12)

With this claim to a patient and quiet spirit, Antonio verbally places himself on a higher moral plane than Shylock, a move that accords with the SCM’s theory that envy is always directed upwards, toward that target the perceiver supposes to be superior. Antonio frames the situation such that he is the superior. It is interesting that Egervari retains this passage in his adaptation (p. 171); therefore, a doomed Jewish prisoner (an incarcerated lawyer), acting the role of Antonio, speaks these words to the “Duke” who is in fact the depraved SS officer who will shortly murder them all, with the exception of “Shylock,” who is murdered by the real Jews.^15^ In this context, the words become even more ironic and meaningful than they are in the Shakespearean text. They signify the moral humanity of the Jewish prisoners, which is also expressed in their dignified behavior throughout.

## What Happens to “Shylock”?

As far as I know, the SCM and BIAS map does not explicitly address what might happen under conditions of stress when a subject targeted by the LW/HC stereotype is perceived to lose competence. Shylock loses his status/competence in *Merchant* and even more strikingly in Egervari’s adaptation, where he is killed. One possibility has to do with the theory that whereas warmth judgments are other-oriented, in that they reflect how others perceive the subject, competence judgments “rebound” on that stereotyped subject (Cuddy et al., 2008, p. 89) – so, in this case, on the commander/“Shylock.” In the world of Egervari’s play, “Shylock” would be perceived as extremely cold, being at the same time – and very confusingly – both a Jew and a Nazi commander with status and murderous (cold to the extreme) intent. On the other hand, in the world of the play within the play, which the commander has insisted they inhabit, at the end of the courtroom scene, Shylock loses his status and his competence. Although there is no stage direction for it in Shakespeare’s script, any director might choose to have Shylock at that point drop the sharpened knife with which he had intended to carve out Antonio’s heart. In Egervari’s adaptation, Gratiano’s verbal attack (4.1.396) is moved to a position immediately preceding and cueing the following climactic action specified by a stage direction:

Shylock is on his knees and the others surround him. As he is reciting his lines, GRATIANO grabs him by the throat and shakes him. Surprised, SHYLOCK drops his knife, which ANTONIO picks up. They all stop for a moment, and then they all jump on top of SHYLOCK. He is killed on the spot before TUBAL has time to get his weapon. (Egervari, 2009, p. 182)

In terms of the SCM, the knife is a crucial resource, and “status assesses the capability of groups to control resources” (Cuddy et al., 2008, p. 94). In the world of the play, at that moment in the action, the “Venetians” have much more status than “Shylock,” who has lost everything. He loses the agency represented by that knife. Competence judgment rebounds immediately on the perceived stereotype/individual, as the model predicts.

A final argument about this moment in the adaptation. When the Nazi commander transforms into the enemy “Shylock,” the world of the play becomes volatile and extremely stressed. The resulting mental conflict in the local environment of the prison on the set is destabilized by profound incongruence between the varied elements of cognitive perception and their affective consequences. The situation can be interpreted using another psychological model, that of affective incoherence, which I understand to be debilitating cognitive-affective incompatibility (Centerbar et al., 2008; Clore and Schnall, 2008).^16^ This instability is a feature of Egervari’s adaptation but not, perhaps, of Shakespeare’s play. Cuddy, Fiske and Glick make the important point that envious stereotypes, such as “Shylock,” although they are ambivalent across the dimensions of warmth and competence, are psychologically consistent for perceivers who do not experience the psychological tension that was classically assumed to be integral to ambivalence (Cuddy et al., 2008. p. 76). With respect to this question, we can observe that in Shakespeare, there is no confusion: all the Venetians are pleased at the outcome, and Shylock falls silent. It is in Auschwitz, however, that the contradiction between the players and roles, and the extreme stress of the war and of the Holocaust, produce explosive tension and confusion at the moment when “Shylock” is killed.

## Summary and Directions for Future Research

I have shown that Shakespeare’s Shylock and Egervari’s post-Holocaust “Shylock” are readable using the SCM and Bias map. My interdisciplinary reading validates the SCM and BIAS map for work in literature, and offers a fresh interpretation of Tibor Egervari’s anti-Nazi Shakespeare adaptation. I hope to have demonstrated the value of interdisciplinary work in such diverse fields of the arts and sciences.

The SCM and BIAS map are a powerful model for understanding the dynamic dance that begins with perception and cognition, then moves to affect and emotion, to sometimes end – at the far right edge of the map – in the worst violence possible, beyond imagining. If the model can help us to discover ways to dissolve a murderous and obdurate ethnic stereotype, that is a goal worth pursuring, as Cuddy, Fiske and Glick themselves affirm (Cuddy et al., 2008, p. 129).^17^

The archival approach developed by Durante et al. (2010) suggests a methodology for investigation of literary heritage by social psychologists. Literature, taking place as it does in the world of the imagination, provides one forum where it is possible to think about the sensitive interface between social stereotypes and social realities such as shared collective memories and demographic shifts (Winiewski and Bulska, 2019).

The SCM shows that warmth judgments are not founded on actual niceness, morality, or warmth, but by the presence or absence of social competition, which may be entirely social and structural; that competency judgments are not determined not by true competency, agency or ability, but by social status, which well be inherited or faked (Cuddy et al., 2008). This consequential finding affirms with evidence what feminists, anti-racists, and others have long known. Yet, as Grigoryev et al. (2019a) have argued, discriminatory treatment of immigrants, for example, produces poor life conditions, which can enhance the negative attitudes of the host population and serve in a sense as self-fulfilling prophecy.

Drawing on his experience as a Hungarian Jewish child caught up in the Nazi Holocaust, Tibor Egervari turned to the ancient theater arts of narrative, costume, mask, make-up, choreography and acting in order to effect the collective transformation of mind of which theater is sometimes capable. His work reminds us of theaters’ precedence in human societies as a means of representing and working through intercultural violence. In Shakespeare’s *A Midsummer Night’s Dream*, the Amazon Queen Hippolyta speaks of theater’s capacity to “transfigure minds all together”^18^ – a psychological notion that suggests a kind of collective cognitive evolution for the better. For millennia, humanity believed in the gravity and importance of the arts. The role of the arts in relation to the evolution of the mind will, I hope, attract more scientific and interdisciplinary study in the future.

## Data Availability Statement

The original contributions presented in the study are included in the article/supplementary material; further inquiries can be directed to the corresponding author.

## Author Contributions

The author confirms that she is the sole contributor to this work and has approved it for publication.

## Conflict of Interest

The author declares that the research was conducted in the absence of any commercial or financial relationships that could be construed as a potential conflict of interest.

## Footnotes

1 For clarity throughout I abbreviate Shakespeare’s *The Merchant of Venice* as (*The) Merchant*, and Egervari’s adaptation, “Shakespeare’s *The Merchant of Venice* in Auschwitz,” as “Merchant.” Likewise, when referring to Egervari’s actor-characters, I add quotation marks, e.g.,“Shylock.”

2 See also Stephen Orgel: “the play has been, as far back as our records take us, a play about Shylock” (Orgel, 2003, p. 159).

3 See also Edinborough’s (1970, p. 459) review of this production, to which Egervari refers in his 2012 essay.

4 It would take another essay to properly address this issue.

5 Goldstein’s (2014) historical article also distinguishes between stereotype and reality, demonstrating that in Shakespeare’s England, Scots were the social group most likely to be negatively stereotyped and targeted as usurers who abstained from pork. In *Post-Colonial Shakespeares*, Martin Orkin cites Shelley Malka’s research disassociating the Jew in *Merchant* from actual Jews living in Venice in the 16th and 17th centuries. Malka “shows … that Jewish representations of the concepts of mercy, justice and revenge are entirely unrelated to the (mis)representation of them in *Merchant”* (Malka, 1996, n.p.; cited in Orkin, 1998, p. 196).

6 Smith notes that her reference to Shakespeare is from Stanley Wells and Gary Taylor, gen. ed., *The Oxford Shakespeare: The Complete Works*, 2^*nd*^ ed., Oxford University Press, 2005.

7 See also Harold Goddard: “Now Shylock is a representative of both […] money, because he himself is a moneylender, and of exclusion, because he is the excluded thing. Therefore the Venetian world makes him their scapegoat” (Goddard, 1951, p. 85).

8 To support this claim, Ackermann cites Eicher (2000, pp. 302–308), Monschau (2010, pp. 19–25 and 68–87), Symington (2005, pp. 244–251), and Bonnell (2008, pp. 119–169); also Márkus (2008, pp. 148–154), Hortmann (1998, pp. 134–137), Endriss (1994, pp. 180–180), Ledebur (1988, pp. 213–218), Drewniak (1983, pp. 250–251), and Wulf (1989, pp. 280–283).

9 The theater department of the National Socialist Ministry of Propaganda (Reichsdramaturgie) adapted the play, eliminating in one way or another the “miscegenation” – the “mixing of Aryan blood with Jewish blood” – represented by Jessica’s marriage to Lorenzo, and deleting Shylock’s monologue in 3.1 perhaps “to evade the universalist humanism that can potentially be seen at work in this speech” (Herman Kroepelen quoted in Eicher, 34; quoted in Ackermann 41; his translation). John O’Connor, in *Shakespearean Afterlives*, also cites Gerwin Strobl on “the matter of Shylock’s daughter marrying an Aryan youth” (Strobl, 1997. n.p.; cited in O’Connor, 2003, p. 131).

10 Ackermann reports on an earlier re-engineering with the opposite, philo-Semitic intent: “Alexander Granach – who, interestingly, had played the role in a production directed by Holl at Berlin’s Volksbühne in 1924 – offers a particularly significant example of the tendency to render or adopt Shylock as a Jewish hero (Ackermann, 2012, p. 42).” In his memoirs, Granach describes his own 1920 production in which he imagined that Shylock moves, after the trial, from Venice to the Ukraine “where he remarries and becomes the progenitor of an entire tribe of Jews: ‘broad-shouldered, hardworking and hungry for new experiences”’ (Granach, 1945, p. 427; cited in Ackermann, 2012, p. 43, his translation). “According to this fantasy, some of Shylock’s offspring became actors and discovered their forefather in the work of Shakespeare’: ‘From their parents and forebears they had learned about Shylock’s story of suffering. Now, on account of their kindred heart, they recognized him. And, leaning on Shakespeare’s genius, they played the character of their ancestor in a tragic and partisan […] manner”’ (Granach, 1945, p. 427; cited in Ackermann, 2012, p. 43, his translation). Theater artist Fritz Kortner, who played Shylock in Reinhardt’s, 1924 *Merchant* in Vienna, wrote in his 1959 memoir of his own ethical, dynamic interpretation of the character (Kortner, 1991; cited in Critchfield, 2008. p. 47, note 1; cited in Ackermann, 2012, p. 44 (Ackermann’s translation).

11 In their Discussion, Grigoryev et al. (2019a) also make the point that the boundaries of “Us” and “Them” are movable and changeable.

12 I cannot at this time identify the Shakespeare text that Egervari, or his translator, Annick Léger, was using. In this paper, therefore, my references to Shakespeare are taken from Shakespeare (2010), *The Merchant of Venice* (2010) edited by John Drakakis, Arden/Bloomsbury.

13 They add: “From an evolutionary perspective, the primacy of warmth makes sense because another’s intent for good or ill matters more to survival than whether the other can act on those goals” (Cuddy et al., 2008, p. 89).

14 As in Hamlet’s famous quibble to Claudio: “A little more than kin, and less than kind.” (Shakespeare, 1987, 1.2.65)

15 As is often the case in Shakespeare productions, actors may take on multiple roles; here the SS officer is both “Tubal” and the “Duke.”

16 This argument is explored in two unpublished conference papers which are available upon request (see Knutson, 2010, 2015).

17 They also observe, “Anti-Semitism has taken the form of an envious prejudice for almost 2000 years.” (p. 127)

HIPPOLYTA But all the story of the night told over, And all their minds transfigured so together, More witnesseth than fancy’s images And grows to something of great constancy; But howsoever, strange and admirable. (Shakespeare, 2008, 5.1.23–27)
